# Genomic language models (gLMs) decode bacterial genomes for improved gene prediction and translation initiation site identification

**DOI:** 10.1093/bib/bbaf311

**Published:** 2025-07-03

**Authors:** Genereux Akotenou, Achraf El Allali

**Affiliations:** Bioinformatics Laboratory, College of Computing, University Mohammed VI Polytechnic, Lot 660, Hay Moulay Rachid, Ben Guerir 43150, Morocco; Bioinformatics Laboratory, College of Computing, University Mohammed VI Polytechnic, Lot 660, Hay Moulay Rachid, Ben Guerir 43150, Morocco

**Keywords:** gene prediction, translation initiation site, large language models, genomic language models, BERT, transformers

## Abstract

Accurate bacterial gene prediction is essential for understanding microbial functions and advancing biotechnology. Traditional methods based on sequence homology and statistical models often struggle with complex genetic variations and novel sequences due to their limited ability to interpret the “language of genes.” To overcome these challenges, we explore genomic language models (gLMs)—inspired by large language models in natural language processing—to enhance bacterial gene prediction. These models learn patterns and contextual dependencies within genetic sequences, similar to how LLMs process human language. We employ transformers, specifically DNABERT, for bacterial gene prediction using a two-stage framework: first, identifying coding sequence (CDS) regions, and then refining predictions by identifying the correct translation initiation sites (TIS). DNABERT is fine-tuned on a curated set of NCBI complete bacterial genomes using a k-mer tokenizer for sequence processing. Our results show that GeneLM significantly improves gene prediction accuracy. Compared with the leading prokaryotic gene finders, Prodigal, GeneMark-HMM, and Glimmer, and other recent deep learning methods, GeneLM reduces missed CDS predictions while increasing matched annotations. More notably, our TIS predictions surpass traditional methods when tested against experimentally verified sites. GeneLM demonstrates the power of gLMs in decoding genetic information, achieving state-of-the-art performance in bacterial genome analysis. This advancement highlights the potential of language models to revolutionize genome annotation, outperforming conventional tools and enabling more precise genetic insights.

## Introduction

Bacterial gene prediction has been a long-standing focus in computational genomics, with numerous tools developed to tackle this challenge. Although significant progress has been made, gene annotation remains an evolving problem, particularly in the accurate identification of coding sequences (CDSs) and translation initiation sites. Traditional gene prediction methods such as Prodigal [1], Glimmer [2], and GeneMark [3] have been widely used for bacterial genome annotation. These tools rely on statistical models, heuristic-based rules, and sequence homology to infer gene structures, but limitations persist. One of the key issues in genome annotation is the variability in composition and organization. High-GC genomes, e.g. pose unique challenges due to an increased number of potential open reading frames (ORFs) and ambiguous start codon selection, leading to a decline in prediction accuracy. Furthermore, annotation of translation initiation site remains a difficult task, as start codon selection is influenced by diverse regulatory mechanisms that vary across species. Although existing tools such as TiCO [4] and TriTISA [5] have been developed to refine translation initiation site (TIS) predictions, these approaches miss several TIS predictions when tested using experimentally verified datasets. Another major challenge lies in the balance between sensitivity and specificity in gene prediction. Traditional methods often overpredict, frequently flagging numerous short ORFs that lack experimental validation. Moreover, genome sequencing analyses have revealed that many essential genes identified through transposon sequencing approaches contain false positives due to genome deletions [6], highlighting the need for methods that improve precision without sacrificing real gene annotations.

Recent advances in artificial intelligence and machine learning have revolutionized sequence analysis by enabling models to extract meaningful features from vast genomic datasets. Several deep learning-based models have been proposed for gene prediction, including convolutional neural networks [7, 8]. Self-supervised learning, a technique successfully applied in natural language processing, has demonstrated remarkable capability in understanding sequential patterns and dependencies. Inspired by this progress, genomic language models (gLMs) have emerged as a promising solution, treating DNA sequences as structured linguistic data to enhance gene annotation [9]. Using deep learning architectures, these models can go beyond traditional sequence alignment, capturing both local and global relationships within DNA sequences. Among these architectures, transformers, particularly Bidirectional Encoder Representations from Transformers (BERT) [10], have shown significant promise in sequence-based tasks. Unlike conventional gene prediction models, which rely on predefined feature sets, BERT-based gLMs dynamically learn sequence representations through self-attention mechanisms [11]. This capability allows them to infer gene structures with greater accuracy by modeling long-range dependencies within bacterial genomes. The application of such models offers a path toward more adaptive and precise genome annotation.

To address these challenges, we introduce a transformer-based gLM suited for bacterial gene prediction. We use a BERT-based architecture to tackle sequence classification tasks in Prokaryotes gene prediction. To address these tasks, we go through a two-stage classification process. As illustrated in Supplementary Figure S1, given a genome, we have many ORFs that can be extracted. Among those ORFs, some of them cover noncoding regions, while others correspond to CDSs. In the first step, we classify the CDSs. Once this is done, in the second step, we focus on the TIS within the coding region. Specifically,

- A BERT-based gLM is developed to identify coding sequence regions and classify translation initiation sites in Prokaryotes genomes.
- The model is initially trained using self-supervised learning in human genomic datasets to learn DNA fragment representations. It is then adapted for gene annotation through a two-stage pipeline: first, a model classifies ORFs into CDS and non-CDS regions, followed by a refinement stage where a second model identifies true TIS sites.
- Comparative evaluations are conducted against traditional gene prediction tools, assessing improvements in precision, recall, and scalability. A case study on verified bacterial genomes is performed to demonstrate the applicability and robustness of our framework in real-world genomic annotation tasks.
- An analysis is performed to interpret the decision-making process of the model, providing insight into its reasoning, an essential aspect of genomic research.

## Materials and methods

### Data collection

To develop a robust and generalized model, it is essential to curate a comprehensive and high-quality dataset. In this study, bacterial genomic data were obtained from the NCBI archive via the GenBank database. Specifically, the assembly summary file was downloaded from the FTP site at ftp.ncbi.nih.gov/genomes/genbank/bacteria. This file, $\sim $1GB in size, provides extensive information on bacterial genomes, including annotations, assembly details, and metadata. To ensure dataset quality, only genomes with complete assembly status were considered and filtering was applied to retain only those classified under the “reference genome” category. This resulted in a dataset comprising 5745 complete and annotated genomes, representing 4823 unique organisms. For each genome in the filtered dataset, two essential files were retrieved: the genome.fna file, which contains the complete nucleotide sequences in FASTA format, and the annotation.gff file, which provides detailed gene annotations, including CDS information. The GFF file delineates genomic features, offering structured annotation data crucial for downstream processing and model training.

### Data processing

To construct high-quality datasets for the translation initiation site and CDS classification tasks, we developed a multi-stage processing pipeline. This pipeline extracts ORFs from bacterial genomes, assigns labels based on genomic annotations, and balances the dataset to ensure robust model training.

To extract ORFs, we scanned the forward and reverse strands of each genome sequence using **ORFipy** [12], a fast and flexible Python-based tool for ORF extraction, to identify potential ORFs based on specific start and stop codons. We filter ORFs that began with one of the start codons (ATG, TTG, GTG, or CTG) and terminated at a stop codon (TAA, TAG, or TGA). Nested overlapping ORFs were retained to provide comprehensive genomic coverage. Each extracted ORF was subsequently assigned a label by comparing its genomic coordinates with the CDS annotations in the corresponding GFF file. We then labeled two types of datasets, one for CDS and the other for TIS classification. The CDS dataset comprises CSV files containing nucleotide sequences with a maximum length of 510, each labeled positive ($1$) or negative. A positive label indicates that the sequence represents the longest truncated ORFs whose start or end positions align with an annotated CDS in the GFF reference file. On the other hand, the TIS dataset includes only sequences from ORFs that match a CDS sequence in the reference file and sequences are assigned a binary label, where $1$ represents a true TIS. To capture sequence context, each TIS-centered sequence includes 30 nucleotides upstream and downstream, resulting in a total length of 60 nucleotides. The overall annotation process of both datasets is illustrated in Supplementary Figure S2.

To ensure class balance and mitigate potential biases during model training, different sampling strategies were applied to the CDS and TIS datasets. For the CDS dataset, negative samples were downsampled based on sequence length to match the distribution of the positive class. This approach increases the difficulty of classification, encouraging the model to learn discriminative features beyond sequence length for distinguishing CDS from non-CDS regions. In contrast, for the TIS dataset, where all sequences have the same fixed length, random undersampling was performed to achieve class balance without introducing additional biases. The dataset was partitioned into training, testing, and evaluation sets. For the CDS dataset, the class-balanced splits consist of 14,975,672 sequences for training, 2,181,188 for testing, and 4,253,562 for evaluation. Similarly, for the TIS dataset, the partitioning resulted in 14,544,028 sequences for training, 2,199,192 for testing, and 4,136,340 for evaluation.

### Tokenization and embeddings

To process DNA sequences effectively during training, we employ the k-mer encoding technique used in the DNABERT [13] framework. In this method, the DNA sequence is split into overlapping substrings of length $k$, known as *k-mers*. Each k-mer serves as a discrete token, like words in natural language processing. According to the DNABERT paper, state-of-the-art performance was achieved using $k=6$. Therefore, for our experimentation, we use a $k=6$ encoding scheme. Given a DNA sequence, the tokenizer splits it into overlapping 6-mer tokens with a stride of 3 for the CDS classification task, while for TIS classification, we use a default stride of 1. After tokenization, each k-mer is transformed into a numerical representation using the pretrained DNABERT model. Each k-mer is mapped to a fixed 768-dimensional vector, corresponding to the hidden size of the BERT [10] architecture. Additionally, we incorporate special tokens: [CLS] at the beginning and [EOS] at the end of each sequence. The [CLS] token provides an additional contextual representation for the sequence as a whole. As a result, for the CDS classification task, each sequence is represented as a matrix of size $(512, 768)$, while for the TIS classification, each sequence is represented as a matrix of size $(62, 768)$. This transfer learning enables the k-mer representations in each sequence to capture meaningful and contextually enriched information. Using DNABERT embeddings, we ensure that the tokenized sequences provide a robust foundation for downstream fine-tuning.

### Model

**Figure 1:**
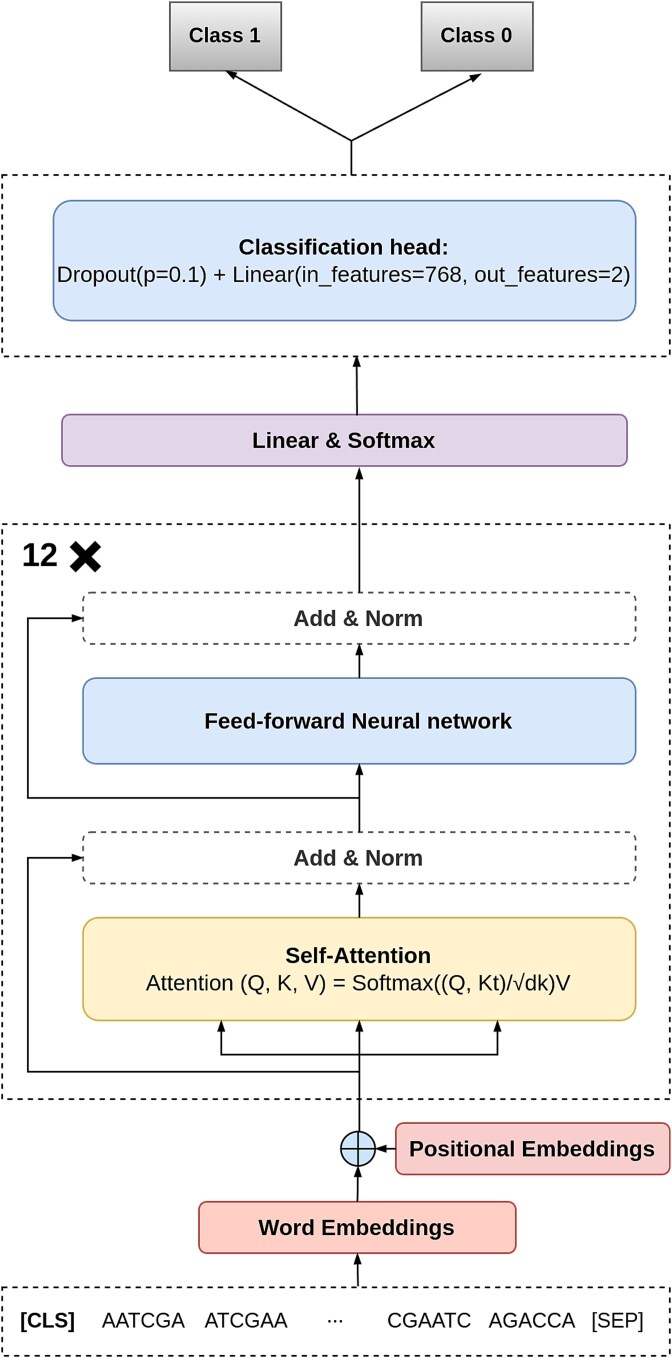
Illustration of the transformer-based BERT architecture used for genomic sequence classification.

Our model is based on the DNABERT framework, which extends the BERT to genomic sequences. DNABERT follows the pretraining and fine-tuning paradigm, where the model first learns general DNA sequence representations and is later fine-tuned for specific downstream tasks. It retains the same transformer-based architecture as BERT, consisting of 12 self-attention layers, 768 hidden dimensions, and 12 attention heads, as shown in Fig. 1.

Given a tokenized input sequence represented as k-mers, the model applies multi-head self-attention to learn contextual relationships across the sequence. The self-attention mechanism is formally defined as


(1) \begin{align*}& \text{MultiHead}(M) = \text{Concat}(\text{head}_{1},..., \text{head}_{h}) W^{O},\end{align*}

where each attention head is computed as


(2) \begin{align*}& \text{head}_{i} = \text{softmax} \left( \frac{M W^{Q}_{i} (M W^{K}_{i})^{T}}{\sqrt{d_{k}}} \right) M W^{V}_{i}.\end{align*}

Here, $ M $ represents the input token embeddings, while $ W^{Q}_{i}, W^{K}_{i}, and\ W^{V}_{i} $ are the learnable query, key, and value matrices for the $ i $th attention head. The self-attention scores determine the contextual relevance of each token concerning all others in the sequence. The final hidden representations from the transformer layers are then used for sequence-level or token-level classification tasks.

### Fine-tuning

**Figure 2:**
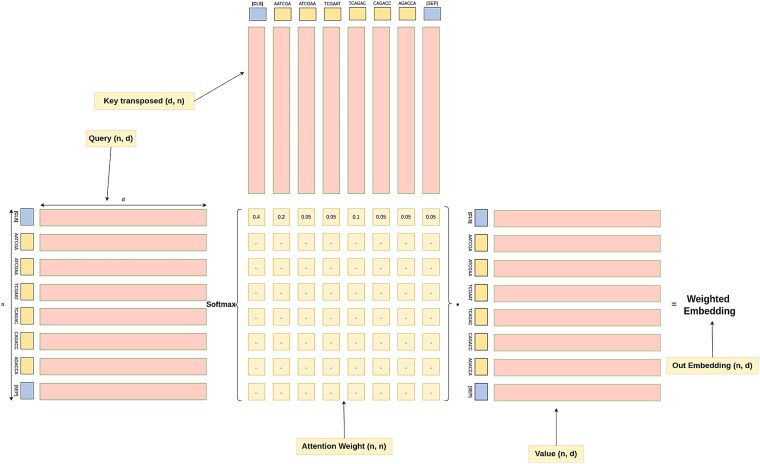
Self-attention mechanism illustrating how attention scores, computed from query, key, and value matrices and normalized via softmax, assign dynamic importance to k-mer tokens to refine sequence embeddings.

For a given sequence, we prepend the classification token [CLS] to the tokenized input sequence and then generate its embedding representation using the DNABERT base model. Self-attention is applied, where the query and key matrices are both derived from the sequence itself. As illustrated in Fig. 2, the attention mechanism computes attention weights using a dot product between the query and key matrices. A softmax function normalizes these weights, and the result is multiplied by the value matrix to adjust the original input embeddings. The re-weighted embeddings are then passed through feed-forward layers, culminating in a classification head appended to the DNABERT base architecture. The final classification decision is based on the embedding of the [CLS] token, which aggregates global sequence features.

Our fine-tuning approach focuses on two key classification tasks: identifying coding sequence regions from DNA sequences and detecting translation initiation sites within CDS regions. Fine-tuning follows the standard BERT-based classification procedure, where the final hidden representation of the [CLS] token is passed through a fully connected layer for sequence-level classification. To enable this, DNABERT is extended by appending a classification head, which consists of the following:

- **Pooler Layer**: aggregates information from the [CLS] token embedding to generate a fixed-size representation for the entire sequence.
- **Fully connected layers**: a feed-forward neural network with one or more layers that maps the pooled representation to the output classes.
- **Softmax Layer**: converts the output logits into probability scores for classification.

The objective function for fine-tuning is the cross-entropy loss, defined as


(3) \begin{align*}& L = - \sum_{i=1}^{N} y_{i}^{\prime} \log(y_{i}),\end{align*}

where $ y_{i}^{\prime} $ represents the ground-truth labels, and $ y_{i} $ denotes the predicted probabilities for each of $ N $ classes. In our case for both experiments $N = 2$.

### Experiment setup

**Figure 3:**
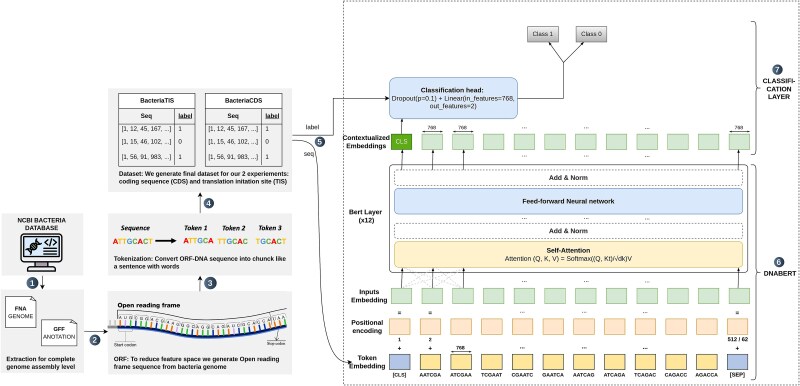
TIS and CDS fine-tuning pipeline illustrating the extraction of ORFs from bacterial genomes, k-mer tokenization, and transformer-based training for genomic region classification, with self-attention capturing long-range dependencies to enhance prediction accuracy.

The entire workflow, from input tokenization and embedding generation to final classification, is summarized in the pipeline diagram (Fig. 3). This diagram outlines all key components, including the DNABERT model, Transformer layers, and the classification head. Given the large-scale dataset, we implement an iterable dataset using PyTorch’s data utilities to efficiently handle data loading and batch processing. The model is trained using the AdamW optimizer with a linear warm-up strategy. The learning rate is initialized at $3e-5$ and gradually decayed to zero over the training steps. The model undergoes fine-tuning on NVIDIA GPUs partitions. To ensure robust evaluation, we partition the dataset into training and testing subsets, where the test set contains only organisms absent from the training set. This experimental design ensures that the model’s performance is assessed on previously unseen taxa, effectively evaluating its generalization ability.

1. **CDS Classification**
  The objective of this task is to classify CDSs in genomic data. The CDS classification experiments were conducted on the Toubkal supercomputer. The training was performed on two NVIDIA A100 GPUs, each equipped with 80GB of memory. Distributed training was implemented across both GPUs with a batch size of 512, and the total training duration was $\sim $37 h for two epochs.
2. **TIS Classification**
  For translation initiation site classification, the objective is to classify 60-bp sequences centered around the potential translation initiation site. This experiment was conducted at the College of Computing, Bioinformatics Lab using a single NVIDIA RTX A5000 GPU with 24GB of memory. The model was trained with a batch size of 768, and the training process completed in $\sim $19 h for three epochs.

### Evaluation metrics

We used a set of standard classification metrics to evaluate the performance of both the individual binary classifiers and the final classifier (via stacking or max-voting). These metrics provide a comprehensive overview of the model’s ability to correctly classify protein transcription factor families. The following metrics were calculated:


(4) \begin{align*} & \text{Accuracy} = \frac{TP + TN}{TP + TN + FP + FN} \end{align*}

(5) \begin{align*} & \text{Precision} = \frac{TP}{TP + FP}\ \end{align*}

(6) \begin{align*} &\qquad\quad \text{Recall} = \frac{TP}{TP + FN} \end{align*}

(7) \begin{align*} & \text{F1 Score} = \frac{2 \cdot \text{Precision} \cdot \text{Recall}}{\text{Precision} + \text{Recall}} \end{align*}

### Post-processing

After TIS prediction, we applied a post-processing step to select the most likely translation initiation site for each predicted coding region. Among all candidate TIS positions within a given ORF group, the site with the highest predicted probability was retained. This decision was recorded using a binary flag (prediction_max_likelihood = 1), marking the optimal site per region. The final annotated results were then formatted into standard outputs, including CSV and GFF files, to ensure compatibility with common genome annotation tools and prepare the data for downstream analysis or visualization.

## Results

This section presents the results obtained from our experiments, structured into three key evaluation stages. First, we report the performance of our model during training and testing, assessing its effectiveness using standard evaluation metrics. Next, we evaluate the model’s performance in a real-world setting by testing it on experimentally verified sequences, providing a practical assessment of its predictive accuracy on biological data validated in the laboratory. Finally, we compare our approach with state-of-the-art gene annotation tools, benchmarking its accuracy and efficiency. These evaluations provide a comprehensive understanding of the model’s capabilities and its potential for practical applications in genome annotation.

### Training performances

In this section, we present the results obtained from training our model across different experiments.

**Figure 4:**
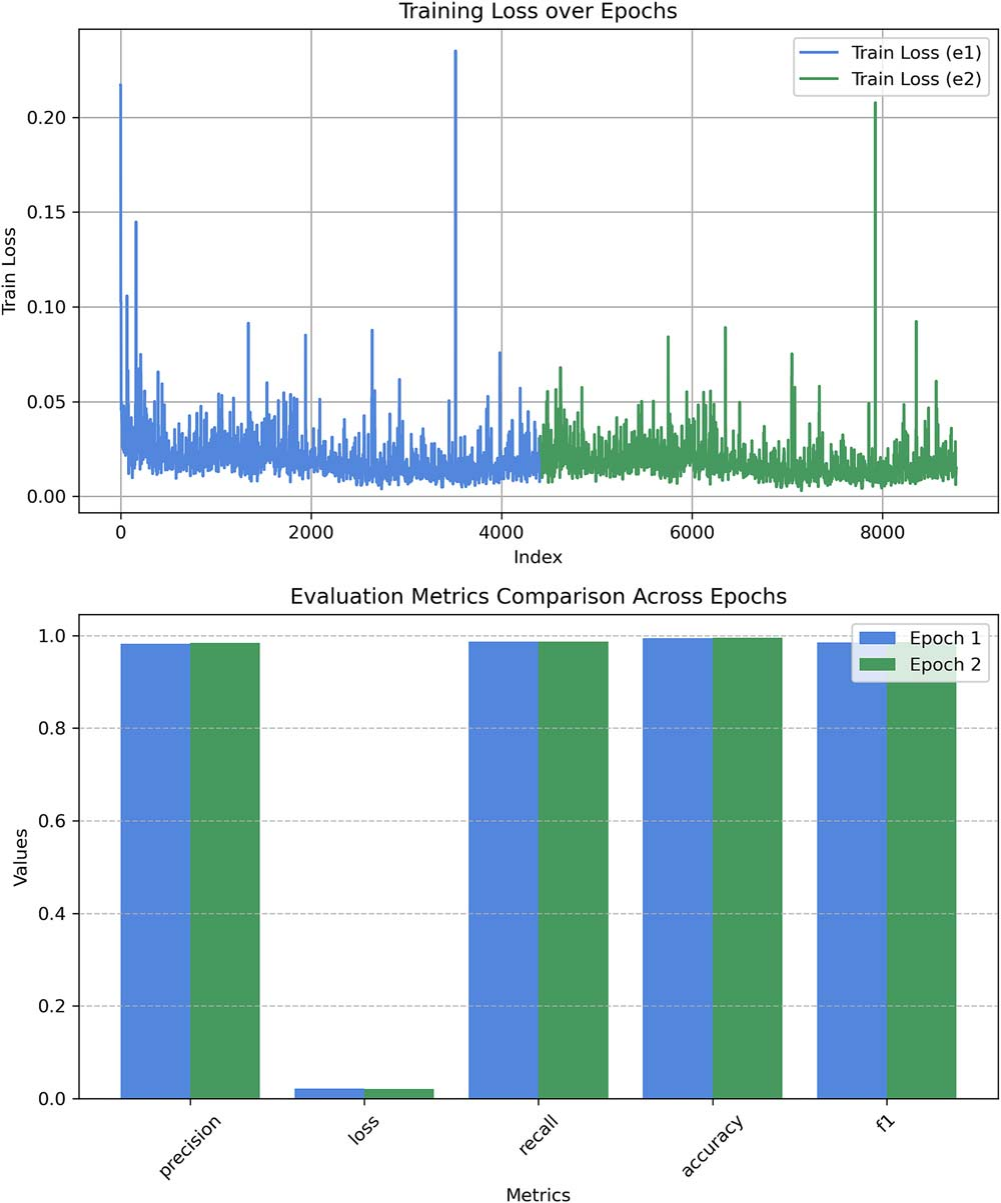
Train and evaluation metrics comparison across epochs for CDS classification experiment. Additional analysis of loss fluctuations is provided in Supplementary Section S3.

**Figure 5:**
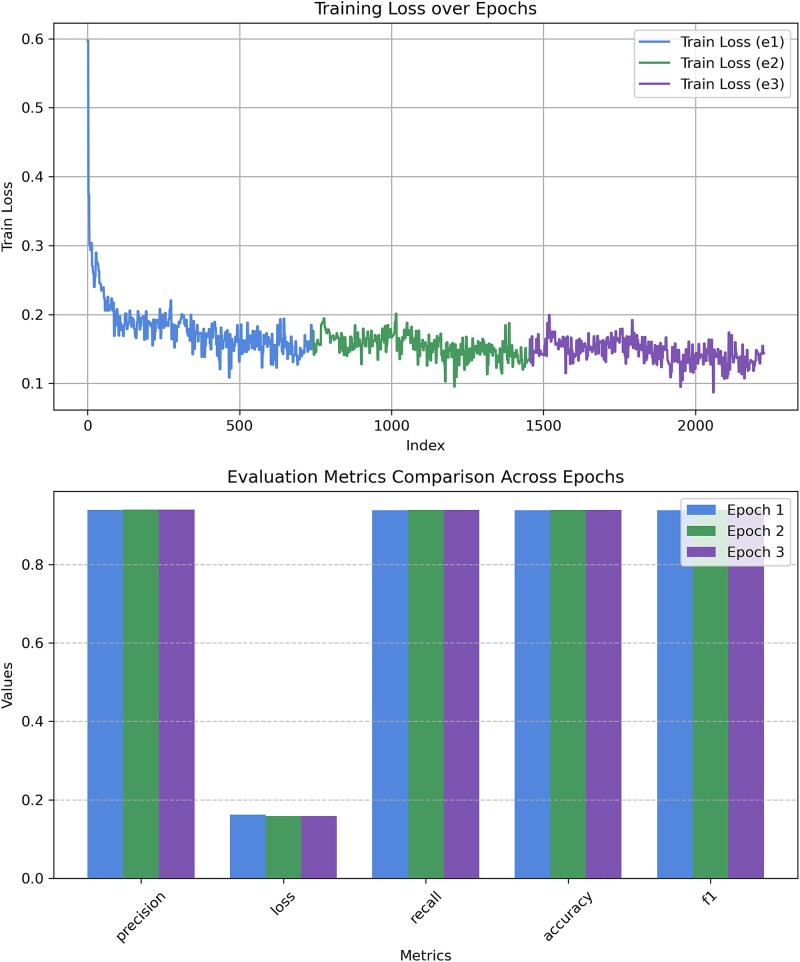
Train and evaluation metrics comparison across epochs for TIS classification experiment.

1. **CDS Classification**
Figure 4 illustrates the training loss progression over multiple steps and the comparison of evaluation metrics across two fine-tuning epochs. Initially, the training loss decreases sharply as the model learns, stabilizing after several thousand iterations. However, sporadic spikes in the training loss were observed, potentially due to the composition of mini-batches with difficult examples. To investigate this, we conducted a reproducibility study by repeating the training with different random seeds and analyzing the loss dynamics. As detailed in Supplementary Sections S3 and S4, the model showed consistent convergence patterns across seeds. We quantified the number of spikes and computed loss distribution statistics, confirming that these fluctuations are not indicative of instability but rather minor stochastic variations in training. Despite these fluctuations, the overall trend shows a consistent reduction in loss, affirming effective learning. After the second fine-tuning epoch, the evaluation metrics showed marginal improvements (Table 1).
  The small gain in performance suggesting that continuing training further might yield diminishing returns or risk overfitting, combined with the computational cost of additional training, led us to halt fine-tuning at epoch 2. Table 1 presents the evaluation metrics for both training epochs and the final test set. The model achieved high classification performance, with precision, recall, and accuracy exceeding 98%. A slight reduction in evaluation loss suggests stable and effective training across epochs. The final model was evaluated on an independent test set, maintaining high accuracy (99.43%) while preserving a low evaluation loss (0.0211). These results confirm the model’s robustness and generalization capabilities.
2. **TIS Classification**
Figure 5 illustrates the training loss trend and evaluation metrics for the TIS classification experiment. Similar to CDS classification, the training loss shows a steep initial decline before stabilizing, with minor oscillations. The evaluation metrics in Table 2 show consistent performance improvements across epochs, with precision, recall, and F1-score increasing slightly over each iteration. However, as seen in the figure, these metrics plateau after the third epoch, showing no further meaningful gains—hence, training was stopped early at epoch 3 to avoid overfitting. The final test evaluation confirmed an accuracy (94.13%) and an evaluation loss (0.1546).
  Given that the TIS annotation used to compute the accuracy is obtained from NCBI which uses gene prediction tools that may not always reflect the ground truth, we further benchmarked our tool against existing methods using all available experimentally verified TIS data for bacterial genomes.

**Table 1:** Evaluation and test set performance metrics for CDS classification

	Evaluation set		Test set
Metric	Epoch 1	Epoch 2	Final score
loss	0.021233	0.020184	0.021132
Precision	0.981846	0.983868	0.983253
F1	0.984364	0.985109	0.984544
Accuracy	0.994265	0.994553	0.994364
Recall	0.986916	0.986357	0.985844

Note: The table presents evaluation metrics for both training epochs and the final test set. The model achieved a classification performance, with precision, recall, and accuracy exceeding 98%. $^{*}$ Test set evaluation confirms the model’s generalization ability with minimal loss and high precision.

**Table 2:** Evaluation and test set performance metrics for TIS classification

	Evaluation set			Test set
Metric	Epoch 1	Epoch 2	Epoch 3	Final score
Loss	0.161598	0.158706	0.156721	0.154614
Precision	0.938289	0.939115	0.939882	0.941508
F1 Score	0.937736	0.938943	0.939723	0.941359
Accuracy	0.937755	0.938949	0.939728	0.941364
Recall	0.937754	0.938948	0.939728	0.941363

Note: The table presents evaluation metrics for both training epochs and the final test set. The model achieved a classification performance, with precision, recall, and accuracy exceeding 93%. $^{*}$ Test set evaluation confirmed generalization ability with a final accuracy of 94.13%.

### Evaluating length bias and overfitting in CDS classification

To ensure the robustness of our CDS classifier and reduce the risk of overfitting to simple features such as ORF length, we implemented a length-aware balancing strategy during training. In the CDS dataset, negative examples were downsampled based on ORF length to match the length distribution of positive (true CDS) examples. This approach was designed to discourage the model from relying on length as a shortcut and to promote learning of discriminative sequence features. To evaluate how well the model generalizes and whether prediction accuracy varies with ORF length, we conducted a length-stratified inference analysis. We used five verified genomes with verified CDS annotations from our benchmarking dataset. We extracted all candidate ORFs, predicted their coding status using the trained model, and compared predictions with ground-truth annotations. ORFs were then grouped into four bins by length, ranging from short (<300 bp) to very long ($\geq $2000 bp). For each bin, we computed precision, recall, and accuracy, and also counted the number of ORFs falling into each length category.

**Figure 6:**
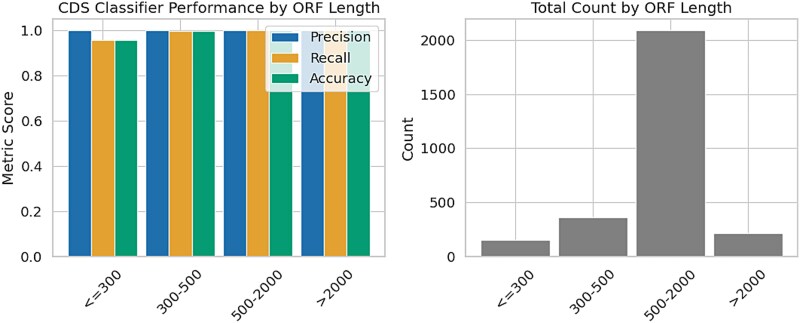
Left: Aggregated CDS classifier performance metrics (precision, recall, accuracy) across ORF length bins using five verified genomes. Right: distribution of ORFs by length.

As shown in Fig. 6, the classifier achieves consistently high performance across all ORF lengths. However, we observe that shorter ORFs, particularly those under 300 bp, are still underrepresented in the test data, which may limit confidence in their performance metrics. Despite this limitation, the overall results provide strong evidence that the model does not rely predominantly on ORF length for classification. This affirms the importance and effectiveness of the length-balancing procedure used during training, which mitigates length-related biases and enhances the biological relevance of learned sequence representations.

### Performance benchmarking on experimentally verified genomes

The effectiveness of GeneLM was benchmarked using experimentally verified CDSs. Benchmarking with real biological data is a standard practice for evaluating gene prediction methods, as seen in prior studies [1, 5, 14]. Building on this, we compared GeneLM with three widely used prokaryotic gene prediction tools: Prodigal [1], GeneMark-HMM [15], and Glimmer3 [16] as well as recent deep learning-based Translation Initiation Site predictors, including TITER [17], DeepGSR [18], and DeepTIS [19]. The evaluation was performed on five bacterial and archaeal genomes—*Escherichia coli*, *Halobacterium salinarum*, *Natronomonas pharaonis*, *Mycobacterium tuberculosis*, and *Roseobacter denitrificans*—selected for their large sets of genes with experimentally verified TISs based on N-terminal peptide sequencing. These organisms (listed in Table 3) had the highest number of annotated TISs, comprising a test set of 2841 verified genes [14].

**Table 3:** Reference clades for the five query species and the sizes of their verified gene test sets, comprising a total of 2,841 genes with start sites confirmed by N-terminal sequencing [14].

Species	**Clade**	**Genomes in clade**	**Verified genes**
*E. coli*	*Enterobacterales*	6311	769
*H. salinarum*	*Archaea*	1125	530
*N. pharaonis*	*Archaea*	1125	282
*M. tuberculosis*	*Actinobacteria*	8097	701
*R. denitrificans*	*Alphaproteobacteria*	4720	526

The reference sequences were obtained from public genome databases corresponding to each species verified annotation dataset [20–24], and were processed through the GeneLM pipeline as well as the comparative tools. All tools were executed using documented and reproducible command-line workflows reflecting best practices for each method (for more details on tool descriptions and execution details see Supplementary Section S5). Performance was measured in terms of correctly predicted TIS positions and the total number of CDSs predicted (see Table 4).

**Table 4:** Comparison with verified annotations showing benchmarking results of traditional TIS annotation tools across five experimentally verified bacterial genomes; metrics include matched TIS (5’+3’ ends and 3’ ends only), with percentages of correctly predicted ends shown in parentheses, and a summary row aggregating performance across all organisms.

**Bacteria**	**GC**	**Verified TIS**	**GeneLM**		**Prodigal v3.0**		**GeneMark-HMM v2.8**		**Glimmer (scratch)**		**Glimmer (iterated)**	
			Matched (5’+3’)	Matched 3’ end	Matched (5’+3’)	Matched 3’ end	Matched (5’+3’)	Matched 3’ end	Matched (5’+3’)	Matched 3’ end	Matched (5’+3’)	Matched 3’ end
E. coli	50.8	769	**744 (96.7%)**	**768 (99.9%)**	338 (44.0%)	345 (44.9%)	595 (77.4%)	759 (98.7%)	276 (35.9%)	366 (47.6%)	319 (41.5%)	369 (48.0%)
H. salinarum	65.7	530	438 (82.6%)	514 (97.0%)	243 (45.8%)	255 (48.1%)	**493 (93.0%)**	**530 (100%)**	220 (41.5%)	266 (50.2%)	220 (41.5%)	265 (50.0%)
M. tuberculosis	65.6	701	**626 (89.3%)**	**695 (99.1%)**	311 (44.4%)	342 (48.8%)	545 (77.7%)	694 (99.0%)	274 (39.1%)	353 (50.4%)	271 (38.7%)	352 (50.2%)
N. pharaonis	63.1	315	248 (78.7%)	302 (95.9%)	169 (53.7%)	176 (55.9%)	**302 (95.9%)**	**314 (99.7%)**	164 (52.1%)	178 (56.5%)	163 (51.7%)	178 (56.5%)
R. denitrificans	58.9	526	**492 (93.5%)**	**523 (99.4%)**	0 (0.0%)	0 (0.0%)	0 (0.0%)	0 (0.0%)	204 (38.8%)	273 (51.9%)	233 (44.3%)	275 (52.3%)
**All genomes**	–	2841	**2548 (89.7%)**	**2802 (98.6%)**	1061 (37.3%)	1118 (39.4%)	1935 (68.1%)	2297 (80.9%)	1138 (40.1%)	1436 (50.5%)	1206 (42.4%)	1439 (50.7%)

**Table 5:** Comparison with GenBank annotations showing the performance of gene-finding algorithms across five bacterial genomes, with each entry reporting the number and percentage of predicted genes matching GenBank annotations at the 3’ end and with full (5’+3’) matches; while not experimentally verified, this benchmark offers a broad snapshot of performance across complete genomes.

**Bacteria**	**GC**	**GenBank TIS**	**GeneLM**		**Prodigal v3.0**		**GeneMark-HMM v2.8**		**Glimmer (scratch)**		**Glimmer (iterated)**	
			Matched (5’+3’) end	Matched 3’ end	Matched (5’+3’)	Matched 3’ end	Matched (5’+3’)	Matched 3’ end	Matched (5’+3’)	Matched 3’ end	Matched (5’+3’)	Matched 3’ end
*E. coli*	50.8	4140	**3767 (91.0%)**	**4033 (97.5%)**	1863 (45.0%)	1968 (47.6%)	3098 (74.8%)	3973 (96.0%)	1474 (35.6%)	2013 (48.6%)	1724 (41.6%)	2026 (49.0%)
*H. salinarum*	65.7	2749	1871 (68.1%)	2559 (93.1%)	1079 (39.3%)	1270 (46.2%)	**2174 (79.1%)**	**2590 (94.2%)**	886 (32.2%)	1244 (45.3%)	900 (32.7%)	1244 (45.3%)
*M. tuberculosis*	65.6	3906	**2664 (68.2%)**	**3709 (95.0%)**	1432 (36.7%)	1904 (48.7%)	2507 (64.2%)	3745 (95.9%)	1251 (32.0%)	1931 (49.4%)	1264 (32.4%)	1939 (49.6%)
*N. pharaonis*	63.1	2820	1978 (70.1%)	2671 (94.7%)	1313 (46.6%)	1461 (51.8%)	**2424 (86.0%)**	**2748 (97.4%)**	1186 (42.1%)	1483 (52.6%)	1217 (43.2%)	1484 (52.6%)
*R. denitrificans*	58.9	4057	**3278 (80.8%)**	**3927 (96.8%)**	0 (0.0%)	0 (0.0%)	0 (0.0%)	0 (0.0%)	1322 (32.6%)	2002 (49.3%)	1528 (37.7%)	2025 (49.9%)
**All genomes**	–	17 672	**13 558 (76.7%)**	**16 899 (95.6%)**	5687 (32.2%)	6603 (37.4%)	10 203 (57.7%)	13 056 (73.9%)	6119 (34.6%)	8673 (49.1%)	6633 (37.5%)	8718 (49.3%)

**Table 6:** Total predicted CDS: comparison of total predicted CDSs across five gene prediction tools for five experimentally verified bacterial genomes, the final row shows the total CDS predictions aggregated across all genomes.

**Bacteria**	**GeneLM**	**Prodigal v3.0**	**GeneMark-HMM v2.8**	**Glimmer (scratch)**	**Glimmer (iterated)**
E. coli	4213	4347	4308	4397	4478
H. salinarum	2659	2851	2762	2717	2762
M. tuberculosis	3853	4204	4029	4240	4349
N. pharaonis	2737	2873	2826	2878	2894
R. denitrificans	4006	4120	4104	4260	4345
**All genomes**	17 468	18 395	18 029	18 492	18 828

Prodigal, GeneMark, Glimmer:  Table 4 summarizes the performance across the five genomes. GeneLM consistently outperformed classical methods across all metrics. Specifically, it achieved the highest number of matched TIS (both 5’ and 3’ ends), with fewer missed predictions. For *E. coli K-12*, GeneLM missed only 25 TIS compared with 431 missed by Prodigal. GeneMark-HMM performed competitively, especially in high-GC genomes such as *M. tuberculosis* and *H. salinarum*, but still lagged behind GeneLM in 5’ start precision. Glimmer3, while historically important, showed weaker performance in both configurations. Its scratch-based model performed worse than the iterated variant, confirming that upstream motif modeling is beneficial, but both approaches underperformed in comparison with GeneLM. A broader benchmark against GenBank annotations is provided in Table 5, highlighting the alignment of predicted genes with known genomic annotations, while Table 6 summarizes the total number of CDS predicted across methods, offering a genome-wide count-based perspective.

TITER, DeepGSR, DeepTIS: in addition to classical gene prediction tools, we also benchmarked GeneLM against recent deep learning approaches from the literature. Since these methods do not provide pretrained models suitable for direct inference, we retrained their architectures on our prokaryotic datasets for the specific task of TIS prediction. Before evaluating them on experimentally verified genomes, we first assessed their performance on a balanced dataset using standard metrics: precision, recall, and F1-score. As shown in Supplementary Figure S5, TITER achieved higher recall (77.05%) but lower precision (66.03%), indicating a tendency to overpredict positive TIS sites. DeepGSR, on the other hand, offered more balanced performance (precision: 71.99%, recall: 73.11%) and a slightly higher F1-score. DeepGSR also required longer training (106.7 h) compared with TITER (84.4 h), likely due to its deeper architecture and larger parameter count. Subsequently, both models were benchmarked alongside GeneLM using experimentally verified TIS datasets across five bacterial genomes (Table 7). Although retrained on the same data for fairness, TITER and DeepGSR consistently underperformed relative to GeneLM. Notably, neither model was able to detect any verified TIS sites in *R. denitrificans*. DeepTIS could not be evaluated due to incomplete architectural and training information, as previously reported by [25].

**Table 7:** Comparison with deep learning approach on TIS prediction tasks, showing the number and percentage of correctly predicted 5’ TIS across five experimentally verified bacterial genomes.

**Species**	**GC**	**Verified TIS**	**GeneLM (5’ end)**	**TITER (5’ end)**	**DeepGSR (5’ end)**
*E. coli*	50.8	769	**744 (96.7%)**	662 (86.1%)	647 (84.1%)
*H. salinarum*	65.7	530	**438 (82.6%)**	391 (73.8%)	395 (74.5%)
*M. tuberculosis*	65.6	701	**626 (89.3%)**	493 (70.3%)	459 (65.5%)
*N. pharaonis*	63.1	315	**248 (78.7%)**	220 (69.8%)	208 (66.0%)
*R. denitrificans*	58.9	526	**492 (93.5%)**	0 (0.0%)	0 (0.0%)
**All genomes**	–	2841	**2548 (89.7%)**	1766 (62.2%)	1709 (60.2%)

Evo2: recent advances in genomic foundation models have introduced Evo2 [26], a long-context DNA language model capable of modeling up to 1 million base pairs. Evo2 was pretrained autoregressively on 8.8 trillion nucleotides across domains of life and is designed for tasks such as sequence generation and zero-shot variant effect prediction. To explore Evo2’s relevance to gene structure modeling, we used the Evo Mechanistic Interpretability Visualizer developed by the Arc Institute and Goodfire. This tool uses a sparse autoencoder trained to decode internal Evo2 activations into discrete, interpretable features. Trained on 100 bacterial genomes, the interpreter reveals features strongly aligned with known genomic concepts such as CDS, tRNA, and secondary structure motifs. We focused on Evo2 feature **f/13606**, which was identified as highly predictive of CDSs. Using its exported activation data, we examined the whole *E. coli* K-12 genome sequence comparing GeneLM prediction with corresponding Evo2 activation values.

**Figure 7:**
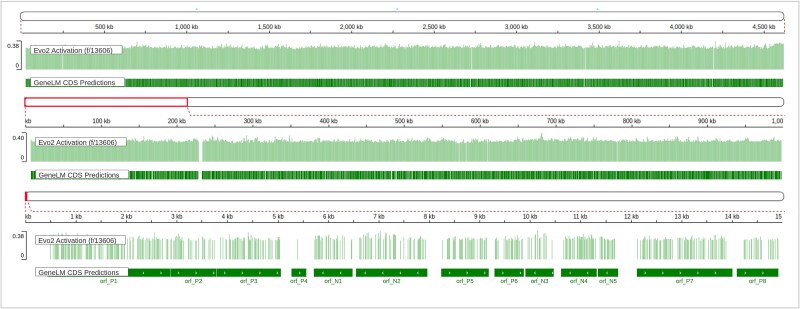
Evo2 activation versus GeneLM CDS predictions on the E. coli genome, with a multi-scale visualization of Evo2 feature activation (f/13606) aligned to GeneLM-predicted CDS regions. Top panel: full genome view showing consistent correspondence between Evo2 activations and GeneLM predictions. Middle panel: 1 Mbp zoom-in highlighting local activation density and refined GeneLM boundaries. Bottom panel: 15 kbp close-up illustrating near-basepair alignment between Evo2 activations and GeneLM-predicted ORFs (Visualization adapted using IGV.js [27, 28]).

As shown in Fig. 7 f/13606 activations consistently overlapped with GeneLM CDS predicted annotations. Notably, Evo2’s unsupervised internal representation showed strong alignment with supervised outputs from GeneLM. These observations suggest that Evo2 feature activations may serve as an effective biological prior or quality-control signal for CDS annotation pipelines. However, we did not include Evo 2 in our benchmarks for several practical and methodological reasons. Evo 2’s hardware requirements are prohibitive for most laboratories: the 7B model already requires a high-memory GPU ($\geq $40GB for full precision), while the 40B variant and context expansion stages rely on H100 GPUs with FP8 support and Vortex or BioNeMo stacks. In contrast, GeneLM operates efficiently on A100 and even A5000-class GPUs, with automatic batch scaling built into our pipeline. Finally, although Evo 2 offers significantly larger context windows, our current results with DNABERT indicate that most prokaryotic CDS regions and surrounding TIS signals fall well within the 512 bp limit. Thus, the benefit of the longer context may be marginal in the prokaryotic setting and comes at significant computational cost. While Evo2 offers compelling interpretability insights, we present it as a complementary tool for understanding genomic signals rather than a direct benchmark competitor, given its distinct design goals and computational constraints. Nevertheless, we acknowledge the potential of Evo 2 to enhance CDS classification and plan to explore its embeddings and context capabilities in future work for hybrid annotation workflows.

### Explaining TIS predictions through attention-based motif visualization and attention-guided sequence disruption

The task of TIS classification involves categorizing 60-nucleotide windows surrounding the TIS site. To understand the model’s decision-making process, we analyze how attention mechanisms contribute to classification. As shown in Supplementary Figures S6 and S7, the model captures distinct patterns through its attention heads. For example, as observed in layer 1, the 11th head focuses on a region $\sim $30 base pairs upstream of the TIS, which may indicate the presence of a promoter site. In contrast, layer 2 exhibits a more selective focus on upstream regions, and a similar pattern is noticeable in layer 9. To derive more generalized insights, instead of analyzing isolated sequences, we extend our visualization across all sequences in our verified test set, which consists of five bacterial species. We focus on layer 11, the final attention layer, as it aggregates all prior learned patterns and directly influences the classification head, playing a crucial role in the final decision. For true TIS sites, we compute attention weights using our TIS prediction model, yielding fixed-size tensors of 11 heads with dimensions $57 \times 57$. The mean attention weights are calculated over all 11 heads per sequence and then averaged across all TIS instances.

**Figure 8:**
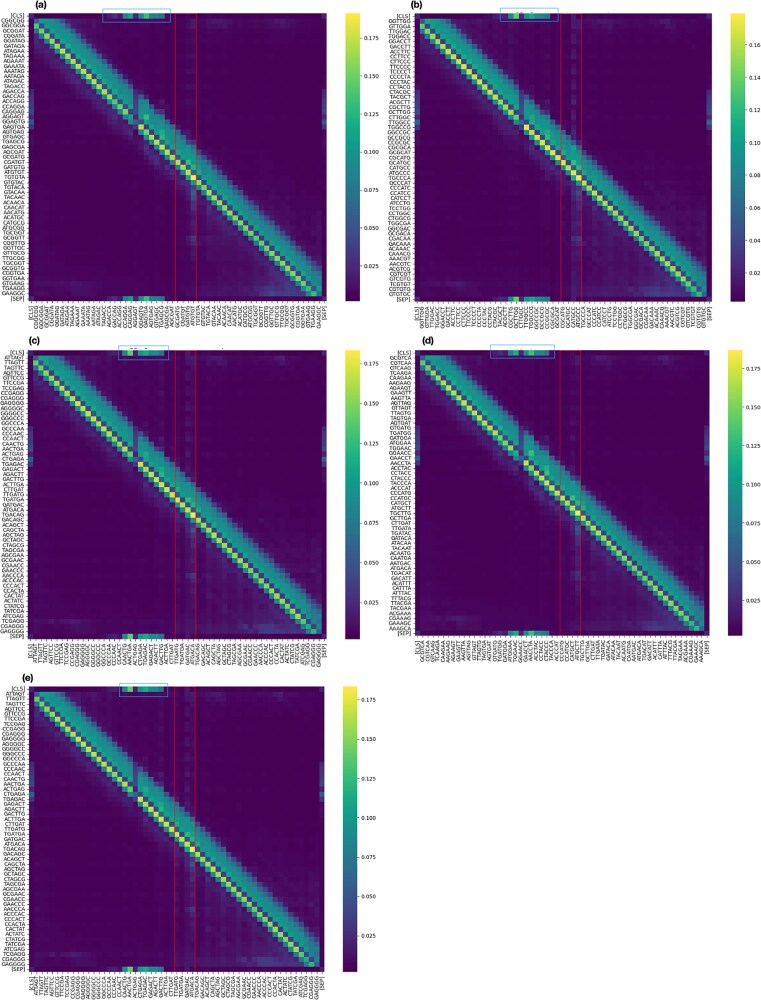
Mean attention weight distribution across verified bacterial species, with each heatmap showing the average attention weights for sequences labeled as true TIS. The visualization highlights the regions most attended to by the classification token [CLS] during final prediction.

Figure 8 presents the attention weight landscape for each bacterial species. The heatmaps labeled **(a)**, **(b)**, **(c)**, **(d)**, and **(e)** correspond to *Escherichia coli*, *Halobacterium salinarum*, *Mycobacterium tuberculosis*, *Natronomonas pharaonis*, and *Roseobacter denitrificans*, respectively. The red box highlights the immediate region surrounding the TIS, while the blue box reveals an intriguing pattern: it marks sequence regions that receive high attention from the classification (CLS) token. These upstream regions may contain potential promoter elements, as the CLS token is responsible for sequence-level classification. This pattern is systematically observed across all bacterial species and aligns with the expected biological significance of promoter regions.

To further assess the biological relevance of the model’s learned attention patterns, we conducted an attention-guided disruption experiment. The aim was to test the sensitivity of TIS prediction scores to perturbations in sequence regions deemed important by the model’s attention weights. Specifically, for each verified TIS-containing sequence, we performed systematic replacements in either high-attention or low-attention regions. The disruption ratio ranged from 0.0 (no modification) to 1.0 (full substitution of the region), and at each ratio, we recorded the model’s TIS prediction probabilities. Two disruption modes were applied: **high attention disruption**, where substitutions targeted positions with the highest attention scores; and **low attention disruption**, where changes were applied to the least-attended positions.

**Figure 9:**
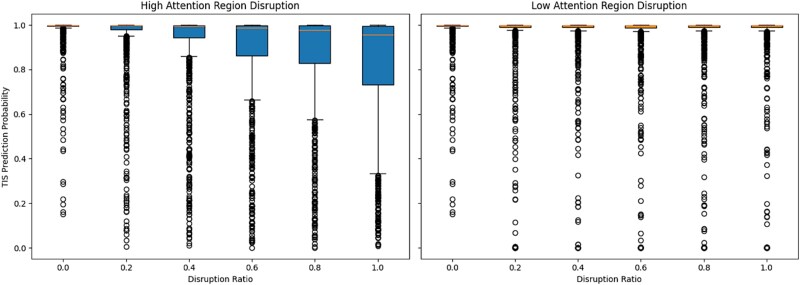
Effect of attention-guided sequence disruption on TIS prediction probabilities. Left: boxplots show predicted probabilities as high-attention regions are increasingly disrupted. Right: boxplots show the impact of disrupting low-attention regions. Each box represents the distribution of predicted TIS probabilities at a specific disruption ratio across all sequences.

As illustrated in Fig. 9, disrupting high-attention regions significantly reduces TIS prediction probabilities as the disruption ratio increases. This suggests that the model heavily relies on the sequence information within these regions when making classification decisions. In contrast, disrupting low-attention regions had little to no impact on prediction scores, with most outputs remaining close to the original probability distribution. These findings validate the model’s ability to attend to biologically informative regions and further support the interpretability of attention mechanisms in genomic sequence classification tasks.

### GeneLM inference scalability and GPU resource analysis

We evaluated the scalability and practical feasibility of GeneLM by benchmarking its inference performance on three bacterial genomes (2.67M to 4.64M bp) across three GPU models with varying memory capacities: NVIDIA A100 (80GB), RTX A5000 (24GB), and RTX A2000 (12GB). Using identical model configurations, we recorded the batch size and total inference time for each GPU–genome combination (Table 8). To maximize efficiency, the GeneLM pipeline automatically scales the batch size based on available GPU RAM, making it highly adaptable across different hardware settings.

**Table 8:** Inference benchmarking of GeneLM across various GPU models and bacterial genomes, with the table reporting GPU memory, batch size, runtime (in minutes), and genome size to illustrate resource requirements and performance scaling.

**Genome**	**GPU name**	**Memory (GB)**	**Batch sze**	**Genome size (bp)**	**Inference time (min)**
bacteria-1	**A100**	79.15	5375	4641 652	21.12
bacteria-2	**A100**	79.15	5375	2668 776	9.75
bacteria-3	**A100**	79.15	5375	4411 532	21.73
bacteria-1	**RTX A5000**	23.66	1556	4641 652	33.08
bacteria-2	**RTX A5000**	23.66	1556	2668 776	15.60
bacteria-3	**RTX A5000**	23.66	1556	4411 532	34.80
bacteria-1	**RTX A2000**	11.65	710	4641 652	101.76
bacteria-2	**RTX A2000**	11.65	710	2668 776	47.74
bacteria-3	**RTX A2000**	11.65	710	4411 532	106.20

As shown in Table 8, GeneLM’s inference time scales with both genome size and GPU memory. On the A100, batch sizes exceed 5000, enabling rapid annotation (under 22 min for genomes over 4.5M bp). The A5000 performs reasonably well ($\approx $33–35 min), while the A2000, constrained by smaller memory and lower batch sizes (710), shows much longer runtimes (up to 106 min). To better compare GPU efficiency across genomes of different lengths, we normalized the inference time by genome size (in Mbp). This yields an average inference speed of $\sim $4.5 min per Mbp for the A100, compared with 7.5 min per Mbp on the A5000 and 22+ min per Mbp on the A2000. These values are derived by dividing the total inference time by the genome length in millions of base pairs (e.g. 21.12 min/4.64 Mbp $\approx $ 4.55 min/Mbp).

This performance gap reflects the memory-bound nature of transformer-based inference: larger batch sizes reduce padding and memory transfers, increasing throughput. Despite this, the model remains usable on mid-range GPUs like the A5000, making it accessible for many research labs.

In future work, inference speed could be further improved by applying techniques such as mixed-precision inference, quantization, or model distillation. These enhancements would help adapt GeneLM for environments with limited computational resources, facilitating broader adoption.

### GeneLM web tool: web application and API

To make GeneLM accessible to a broader community of researchers and bioinformaticians, we developed a web-based genome annotation interface and a RESTful API. These tools serve as practical applications of the model, allowing users to annotate bacterial genomes using GeneLM without requiring deep computational expertise. The tool is publicly available on GitHub at github.com/Bioinformatics-UM6P/GeneLM.

GeneLM web tool supports two modes of input: direct input where users can paste a genome sequence into the provided text area or file upload where users can upload a FASTA file for processing. Once the input is provided, users can specify the desired output format, selecting either GFF or CSV. Upon submission, the system processes the annotation and generates structured output files. The user-friendly interface ensures accessibility for researchers and bioinformaticians. Supplementary Figures S8 and S9 show a snapshot of the interface and an example of the downloadable annotated output, respectively.

Beyond the graphical interface, we developed a RESTful API to enable programmatic access for flexible genome annotation. The API allows users to submit annotation tasks asynchronously, queue multiple annotation jobs efficiently, track annotation progress, retrieve annotated results, and cancel annotation tasks if necessary. The API is well-documented, providing multiple endpoints for annotation submission, result retrieval, and task management. This ensures seamless integration into bioinformatics pipelines and allows users to perform genome annotation directly through Python scripts or other computational workflows. This integrated web-based and API-driven annotation pipeline provides an efficient, scalable, and user-friendly solution for genome annotation, bridging the gap between machine learning-based gene prediction and real-world biological research.

## Discussion and conclusion

This work demonstrates the potential of transformer-based gLMs for accurate and interpretable bacterial gene annotation. Built upon the DNABERT architecture, GeneLM introduces a biologically structured two-stage classification pipeline that separates the tasks of CDS identification and TIS refinement. This modular design aligns with biological gene structure and contributes to enhanced prediction precision across diverse bacterial species.

A key strength of GeneLM lies in its ability to generalize across a wide range of organisms while maintaining high accuracy and recall. This is supported by a carefully curated and balanced dataset, including length-stratified sampling to mitigate ORF length bias. Qualitative and quantitative analyses of self-attention maps further revealed that the model consistently focuses on biologically meaningful upstream regions near TIS sites, suggesting sensitivity to promoter-like motifs. These insights were reinforced by attention-guided sequence disruption experiments, which demonstrated that perturbing high-attention regions significantly alters the model’s predictions.

Although the model is initially pretrained on human genomic sequences to leverage large-scale self-supervised learning, we acknowledge the substantial evolutionary and organizational differences between eukaryotic and prokaryotic genomes. To address this domain gap, GeneLM is fine-tuned extensively on a large and diverse bacterial dataset, enabling adaptation to prokaryotic-specific features. The strong performance observed across multiple bacterial clades—including those with high-GC content—and the benchmarking against experimentally verified datasets provide evidence that this adaptation is effective in practice.

In benchmarking experiments using experimentally verified genomes, GeneLM achieved state-of-the-art performance in recovering annotated TIS positions and reducing false positives compared with classical gene prediction tools like Prodigal, GeneMark-HMM, and Glimmer, as well as recent deep learning approaches. Additionally, our evaluation of inference scalability across three GPU tiers shows that GeneLM adapts batch size based on available memory, enabling efficient deployment even on mid-range hardware. To further increase accessibility, we release GeneLM as an open-source web tool and API to facilitate its integration into existing annotation workflows. While transformer-based models impose higher computational demands than heuristic methods, future directions include mixed-precision inference, quantization, and model distillation to reduce resource usage.

In summary, GeneLM provides a reproducible and interpretable framework for bacterial gene boundary identification. Its contributions span task formulation, dataset design, biological insight, and deployment readiness—offering a solid foundation for further advancements in automated genome annotation and potential extensions toward noncoding and regulatory region discovery.

**Key Points**

- A BERT-based genomic language model is developed to identify coding sequence regions and classify translation initiation sites in Prokaryotes genomes.
- The model is initially trained using self-supervised learning on human genomic datasets to learn DNA chunk representations. It is then adapted for gene annotation through a two-stage pipeline: first, a model classifies ORFs into CDS and non-CDS regions, followed by a refinement stage where a second model identifies true TIS sites.
- Comparative evaluations are conducted against traditional gene prediction tools, assessing improvements in precision, recall, and scalability. A case study on verified bacterial genomes is performed to demonstrate the model’s applicability and robustness in real-world genomic annotation tasks.

## Supplementary Material

Supplementary_Materials_bbaf311

## Data Availability

The current implementation of our work and the trained models and their weights are available at github.com/Bioinformatics-UM6P/GeneLM.
