# Supplementary material for: Genomic language models (gLMs) decode bacterial genomes for improved gene prediction and translation initiation site identification
Source: Brief Bioinform. 2025 Jul 3;26(4):bbaf311. doi: 10.1093/bib/bbaf311 (PMC12222049; doi:10.1093/bib/bbaf311)
Supplement: Supplementary_Materials_bbaf311 [file supplementary_materials_bbaf311.pdf]

Supplementary Materials

Genomic Language Models (gLMs) decode bacterial genomes for improved Gene Prediction and Translation Initiation Site identification

Genereux Akotenou, Achraf El Allali*
Bioinformatics Laboratory, College of Computing, Mohammed VI Polytechnic University, Lot 660, Hay Moulay Rachid, Ben Guerir 43150, Morocco

Contents

S1. Illustration of Translation Initiation Site and Coding Sequence zones in the genome 2
S2. Overview of the dataset labeling pipeline for CDS and TIS classification. 3
S3. Reproducibility Analysis Across Multiple Random Seeds (1) 4
S4. Reproducibility Analysis Across Multiple Random Seeds (2) 5
S5. Benchmarking GeneLM against GeneMark-HMM, Prodigal, Glimmer3, and Deep Learning Approaches 5
S6. Explaining TIS Predictions Through Attention-Based Motif Visualization 7
S7. Observed pattern between the CLS token and TIS upstream positions 8
S8. GeneLM Web Tool: Web Application and API Interface 9
S9. GeneLM Web Tool: Generated GFF Output file 9

*Corresponding author: achraf.elallali@um6p.ma

S1. Illustration of Translation Initiation Site and Coding Sequence zones in the genome

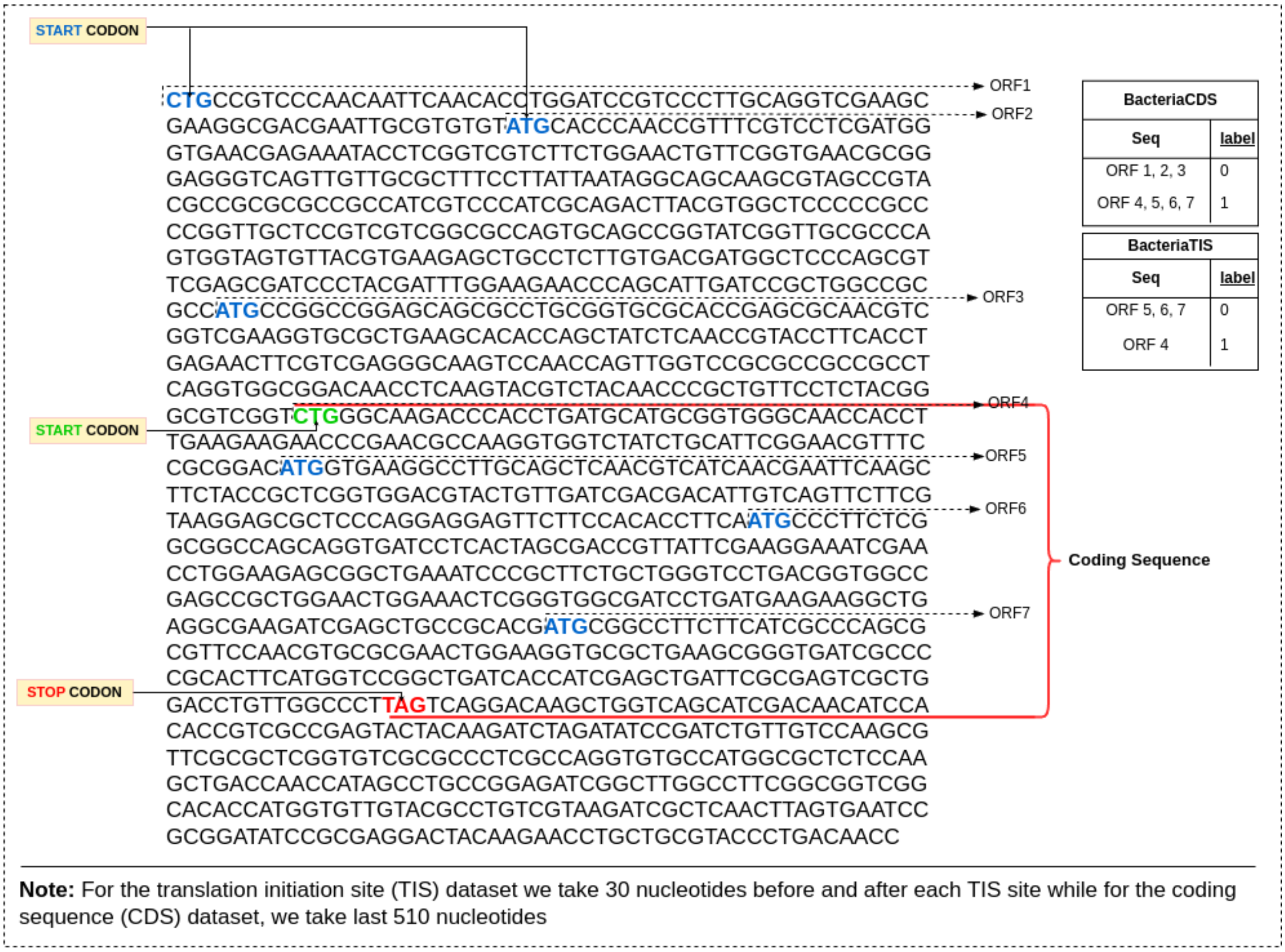

Supplementary Figure S1: Illustration of Translation Initiation Site and Coding Sequence zones in the genome.

S2. Overview of the dataset labeling pipeline for CDS and TIS classification.

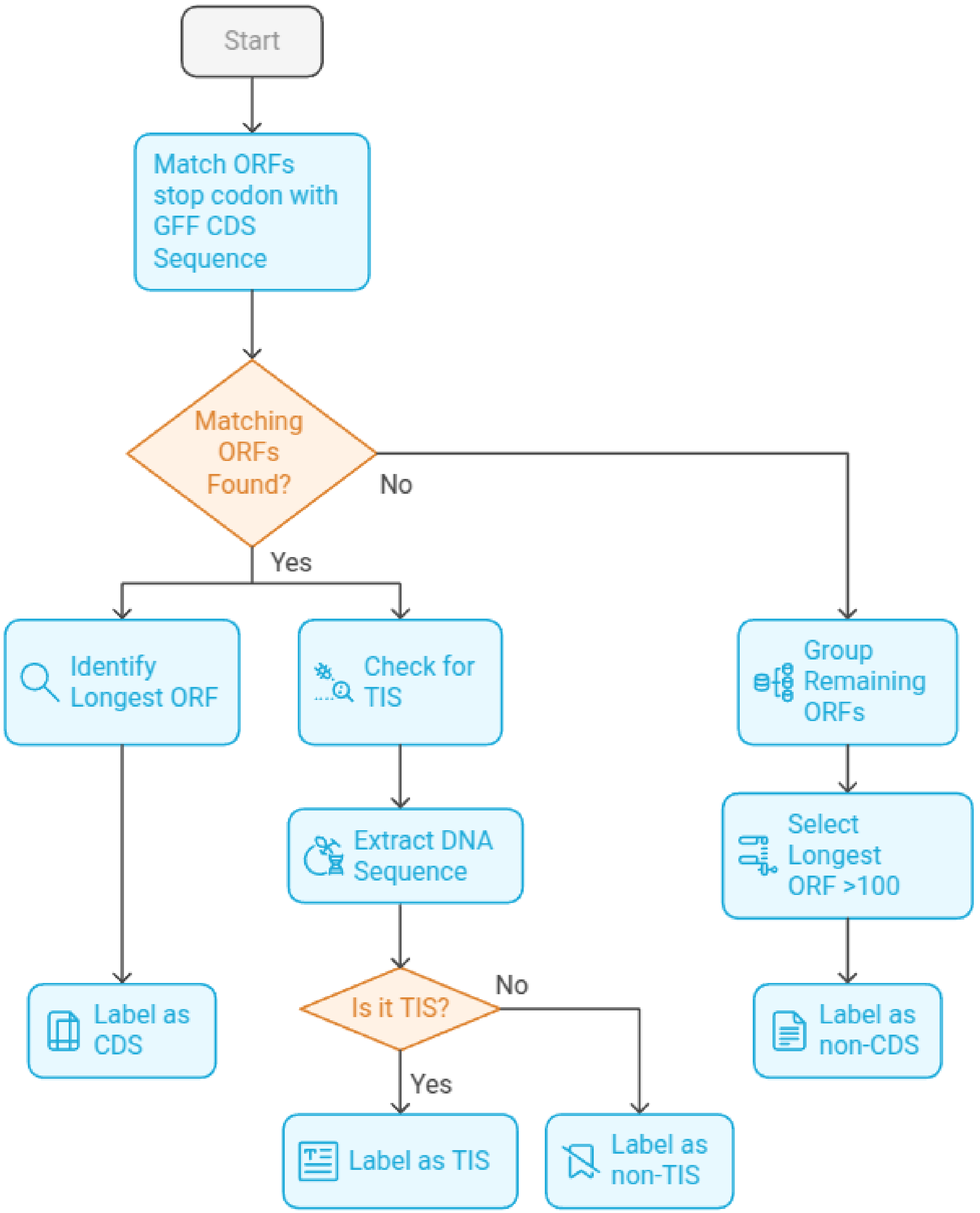

Supplementary Figure S2: Overview of the dataset labeling pipeline for CDS and TIS classification. The pipeline involves extracting nucleotide sequences, assigning binary labels based on alignment with annotated CDS regions in the GFF reference file, and structuring the sequences for downstream analysis.

S3. Reproducibility Analysis Across Multiple Random Seeds (1)

To assess the reproducibility and robustness of our CDS classification model, we repeated the training using three random seeds: 42, 123, and 2025. This analysis evaluates the stability of convergence and generalization performance under varying initializations.

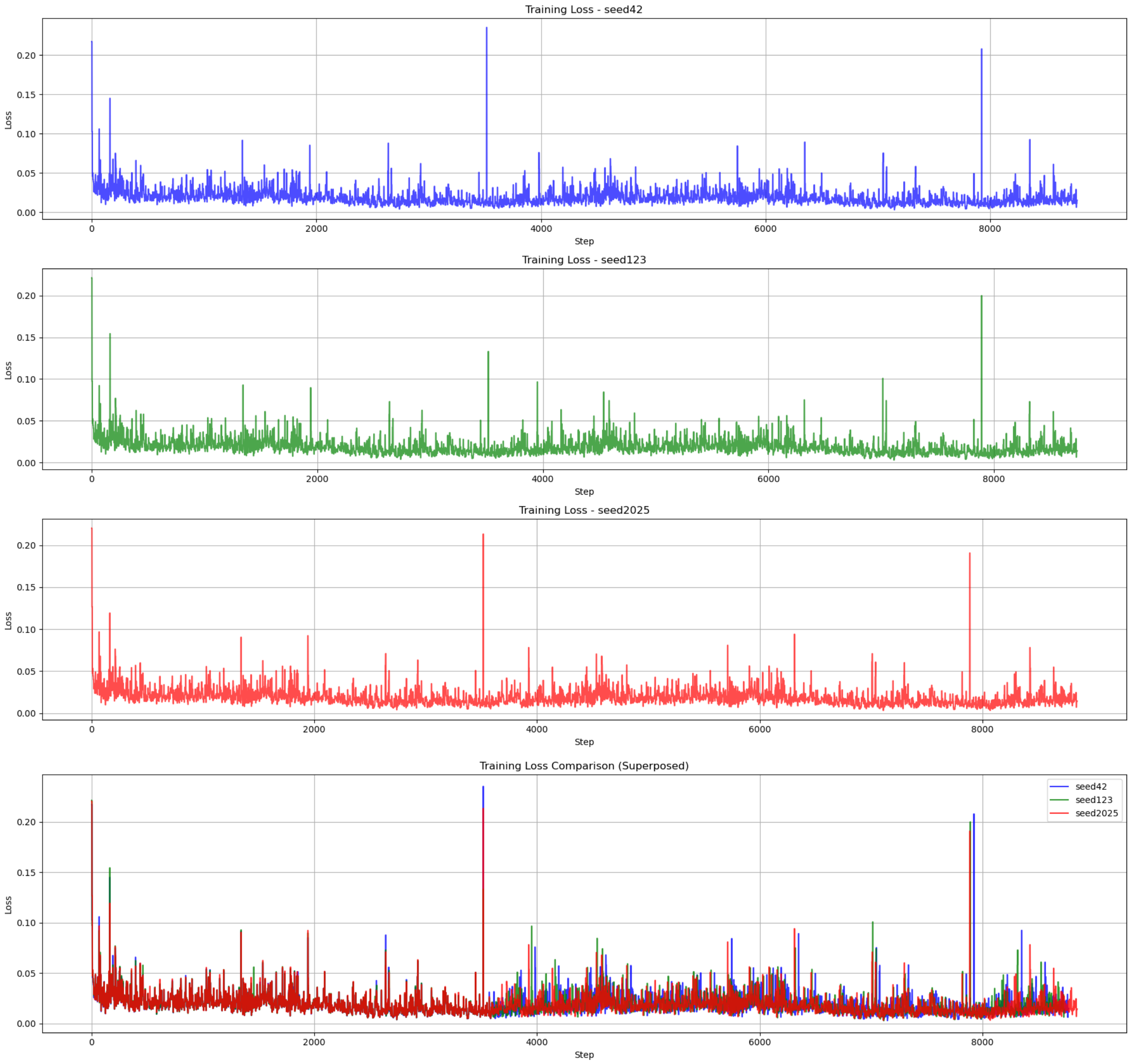

Supplementary Figure S3: Training loss comparison for 3 independent training runs using different random seeds (42, 123 and 2525). Both exhibit similar convergence behavior and occasional spikes, indicating consistent optimization patterns. This confirms that the spikes are due to the composition of mini-batches with difficult examples.

S4. Reproducibility Analysis Across Multiple Random Seeds (2)

Supplementary Table S4: Evaluation metrics and training loss statistics across random seeds.

(a) Evaluation metrics across epochs grouped by random seed.

Metric	Seed 42		Seed 123		Seed 2025	
	E1	E2	E1	E2	E1	E2
Loss	0.02123	0.02018	0.02015	0.02029	0.02037	0.02020
Precision	0.98185	0.98387	0.98223	0.98362	0.98360	0.98321
Recall	0.98692	0.98636	0.98772	0.98593	0.98555	0.98668
F1	0.98436	0.98511	0.98496	0.98477	0.98457	0.98494
Accuracy	0.99427	0.99455	0.99446	0.99443	0.99436	0.99448

(b) Training loss dynamics across seeds.

Seed	Mean Loss	Std. Dev.	Spikes (#)	Max Loss	Min Loss
42	0.0193	0.0124	25	0.2350	0.0030
123	0.0195	0.0120	28	0.2212	0.0029
2025	0.0193	0.0120	22	0.2202	0.0033

Note: We further analyzed loss stability by computing statistics on the full loss curve. ”Spikes” are defined as points exceeding 2× the local standard deviation above a rolling average. This helps identify outliers due to batch composition or optimization variance. Despite occasional spikes, the loss distributions remain consistent across seeds.

These results suggest that although individual training runs exhibit some stochastic variation, the model is highly reproducible across seeds in terms of convergence and generalization.

S5. Benchmarking GeneLM against GeneMark-HMM, Prodigal, Glimmer3, and Deep Learning Approaches

Tool Descriptions and Execution Details

GeneLM: GeneLM is our transformer-based genomic language model designed for bacterial gene prediction and Translation Initiation Site (TIS) identification. It uses DNABERT embeddings with 6-mer tokenization in a two-stage pipeline: first detecting coding sequences from ORFs, then refining TIS positions within them. Trained on over 5,700 complete bacterial genomes, GeneLM leverages attention mechanisms to capture regulatory patterns around start sites.

Prodigal: Prodigal is a widely used gene prediction tool optimized for prokaryotic genomes. It identifies genes based on coding potential and translation initiation models. In our benchmark, Prodigal v3.0.0-rc.1 was installed from github.com/hyattpd/Prodigal and executed using the following command that generate file we convert latter to GFF format:

```
prodigal -i sequence.fasta -o prodigal.genes
```

GeneMark-HMM: GeneMark-HMM v2.8 is a probabilistic gene prediction tool based on hidden Markov models (HMMs), incorporating species-specific codon usage and heuristic training. It is part of the GeneMark suite, available at genemark.bme.gatech.edu. In our benchmark, we used the latest release of the suite and followed its recommended pipeline. After detecting the GC content of the genome using

the `gc` utility, we selected the appropriate pre-trained model (e.g., `heu_11_50.mod`) provided with the tool to match the genome's GC profile. The prediction steps were executed as follows:

```
gmsn.pl sequence.fasta --species Ecoli --clean
gc sequence.fasta
gmhmmp -m heu_11_50.mod -f G -o genemark-hmm.gff sequence.fasta
```

Glimmer3 (from scratch and iterated): Glimmer3 is based on interpolated Markov models trained on long open reading frames (ORFs). We use latest version of Glimmer3 v3.02 that we installed from ccb.jhu.edu/software/glimmer. We ran two configurations: **From Scratch mode**: trains directly from long ORFs in the genome and **Iterated mode** which refines predictions using initial predictions as training data.

```
# Glimmer-from-scratch
cd Ecoli && csh g3-from-scratch.csh sequence.fasta  from-scratch
# Glimmer-iterated
cd Ecoli && csh g3-iterated.csh sequence.fasta iterated
```

TITER: TITER [5] predicts TIS from genomic sequences using a hybrid convolutional and recurrent neural network, integrating codon preference priors. For this study, we reimplemented and trained it on bacterial genomes for fair comparison.

DeepGSR: DeepGSR [2] applies an optimized deep learning architecture to recognize genomic signals, using a convolutional backbone. We adapted and retrained it using our prokaryotic dataset.

DeepTIS. DeepTIS [4] uses a two-stage architecture combining Content-RCNN and Integrated-CNN modules. However, due to incomplete architecture details and missing reproducibility elements, we were unable to benchmark this model, in line with similar limitations noted in Clauwaert et al. [1].

Implementation and Reproducibility. All tools were executed using the most recent stable versions, with commands and configuration scripts stored in the GeneLM GitHub repository under benchmark/-TOOL/.

	True Positives	True Negatives	False Positives	False Negatives	Precision	Recall	Specificity	F1 Score	Training Duration (h)
TITER	2189.000000	1715.000000	1126.000000	652.000000	66.03%	77.05%	60.37%	71.12%	84.4
DeepGSR	2077.000000	2033.000000	808.000000	764.000000	71.99%	73.11%	71.56%	72.55%	106.7

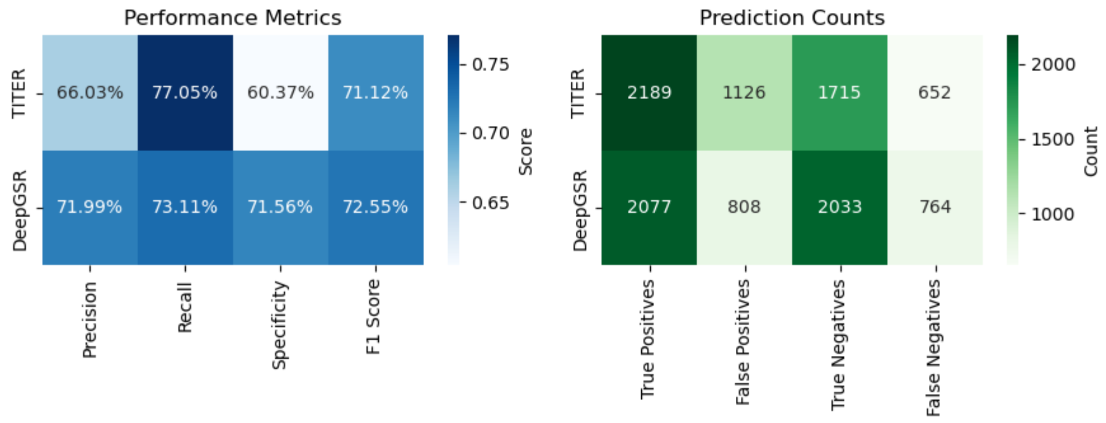

Supplementary Figure S5: Evaluation of TIS prediction performance for TITER and DeepGSR. Left: Heatmap of classification metrics (Precision, Recall, Specificity, F1 Score). Right: Heatmap of raw prediction counts (True Positives, False Positives, True Negatives, False Negatives). Results highlight the trade-offs between sensitivity and precision in each model.

S6. Explaining TIS Predictions Through Attention-Based Motif Visualization

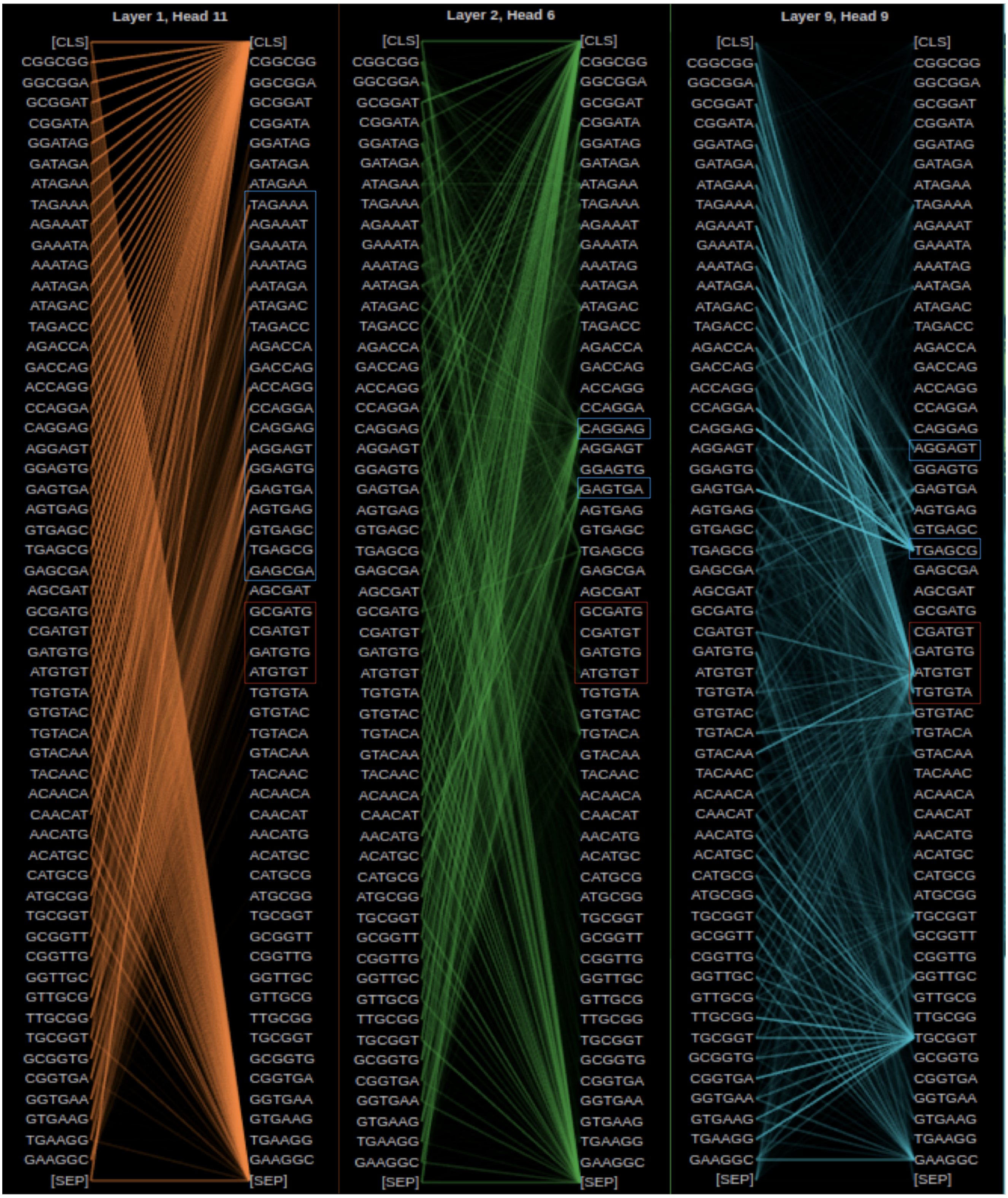

Supplementary Figure S6: Visualization of attention mechanisms in the TIS classifier generated with BertViz tool [3].

S7. Observed pattern between the CLS token and TIS upstream positions

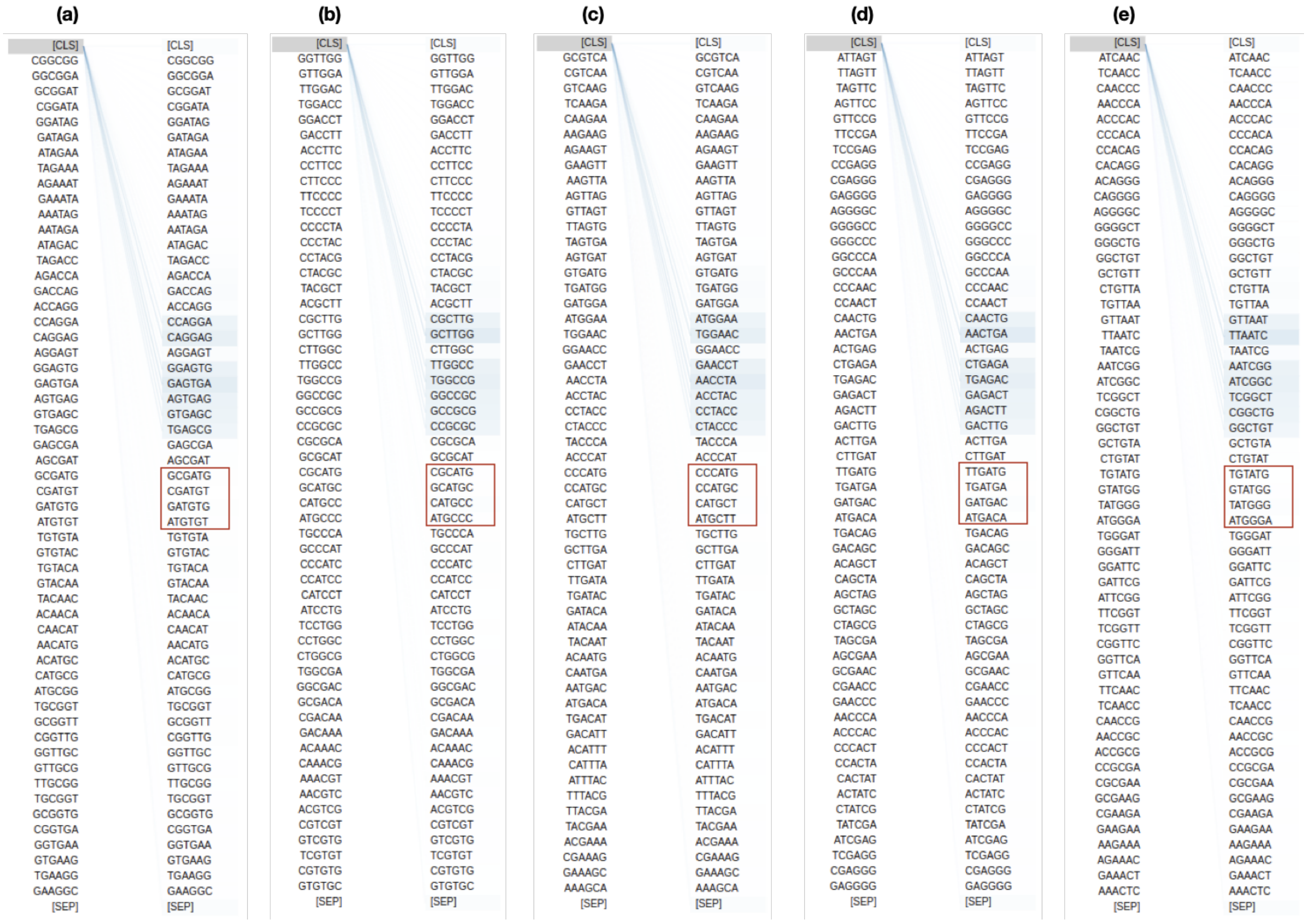

Supplementary Figure S7: Observed systematic pattern between the CLS token and TIS upstream positions for a sample sequence.

S8. GeneLM Web Tool: Web Application and API Interface

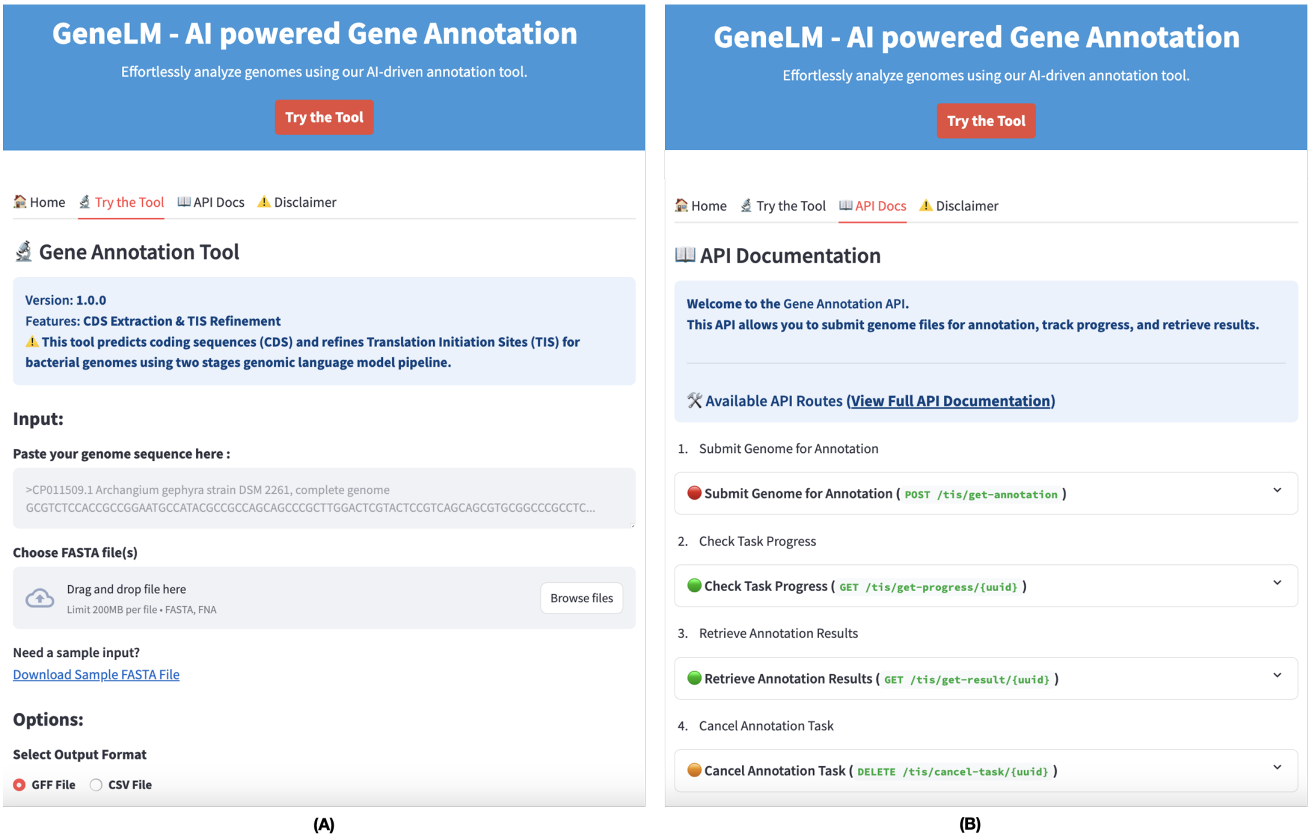

Supplementary Figure S8: Web interface for genome sequence annotation. (A) The Gene Annotation Tool interface allows users to input genome sequences by pasting them or uploading FASTA files. It provides CDS extraction and TIS refinement functionalities with options to select the output format (GFF or CSV). (B) The API Documentation interface outlines available API routes for genome annotation, including submitting sequences, tracking progress, retrieving results, and canceling tasks.

S9. GeneLM Web Tool: Generated GFF Output file

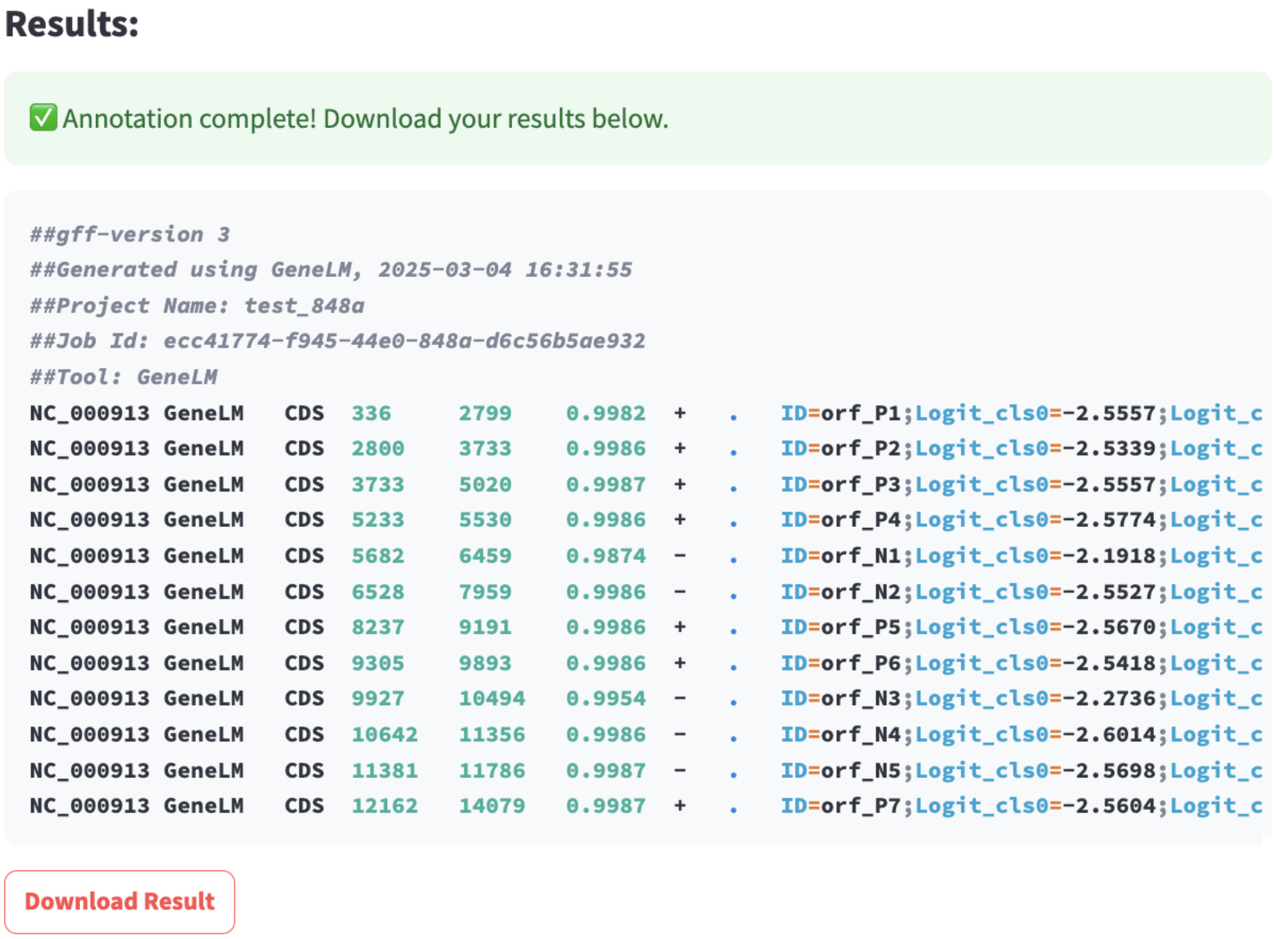

Supplementary Figure S9: Example of a generated GFF output file showing the annotation results, including sequence IDs, feature types, start and end positions, strand orientation, confidence scores, and additional metadata.

References

[1] Jim Clauwaert, Zahra McVey, Ramneek Gupta, and Gerben Menschaert. Tis transformer: remapping the human proteome using deep learning. *NAR Genomics and Bioinformatics*, 5(1):lqad021, 2023. doi: 10.1093/nargab/lqad021. URL https://doi.org/10.1093/nargab/lqad021.

[2] Manal Kalkatawi, Arturo Magana-Mora, Boris Jankovic, and Vladimir B. Bajic. Deepgsr: an optimized deep-learning structure for the recognition of genomic signals and regions. *Bioinformatics*, 35 (7):1125–1132, 2019. doi: 10.1093/bioinformatics/bty752.

[3] Jesse Vig. A multiscale visualization of attention in the transformer model. In *Proceedings of the 57th Annual Meeting of the Association for Computational Linguistics: System Demonstrations*, pages 37–42, Florence, Italy, July 2019. Association for Computational Linguistics. doi: 10.18653/v1/P19-3007. URL https://www.aclweb.org/anthology/P19-3007.

[4] Chao Wei, Junying Zhang, and Yuan Xiguo. Deeptis: Improved translation initiation site prediction in genomic sequence via a two-stage deep learning model. *Digital Signal Processing*, 117:103202, 2021. doi: 10.1016/j.dsp.2021.103202. URL https://www.sciencedirect.com/science/article/pii/S1051200421002414.

[5] Sai Zhang, Hailin Hu, Tao Jiang, Lei Zhang, and Jianyang Zeng. Titer: predicting translation initiation sites by deep learning. *Bioinformatics*, 34(4):516–524, 2018. doi: 10.1093/bioinformatics/btx682.
